# Multi-Step Two-Dimensional Ultrasonic-Assisted Grinding of Silicon Carbide: An Experimental Study on Surface Topography and Roughness

**DOI:** 10.3390/mi15070915

**Published:** 2024-07-15

**Authors:** Hongbo Li, Tao Chen, Wenbo Bie, Fan Chen, Yuhao Suo, Zhenyan Duan

**Affiliations:** 1 School of Intelligent Manufacturing and Electrical Engineering, Nanyang Normal University, Nanyang 473061, China; 2 School of Mechanical and Electronic Engineering, Wuhan University of Technology, Wuhan 430070, China; chent29@whut.edu.cn (T.C.); suoyuhao001@sina.com (Y.S.); 305095@whut.edu.cn (Z.D.); 3 School of Electrical and Mechanical Engineering, Pingdingshan University, Pingdingshan 467000, China; wenbo187120@163.com (W.B.); 13403917708@163.com (F.C.)

**Keywords:** two-dimensional ultrasonic-assisted grinding, silicon carbide, surface roughness, surface micro-morphology, surface damage

## Abstract

Two-dimensional ultrasonic-assisted grinding (2D-UAG) has exhibited advantages in improving the machining quality of hard and brittle materials. However, the grinding mechanism in this process has not been thoroughly revealed due to the complicated material removal behaviors. In this study, multi-step 2D-UAG experiments of silicon carbide are conducted to investigate the effects of machining parameters on surface quality. The experimental results demonstrate that the tool amplitude and the workpiece amplitude have similar effects on surface roughness. In the rough grinding stage, the surface roughness decreases continuously with increasing ultrasonic amplitudes and the material is mainly removed by brittle fracture with different surface defects. Under semi-finishing and finishing grinding steps, the surface roughness first declines and then increases as the tool amplitude or workpiece amplitude grows from 0 μm to 8 μm and the inflection point appears around 4 μm. The surface damage contains small-sized pits with band-like distribution and localized grooves. Furthermore, the influences of cutting parameters on surface quality are similar to those in conventional grinding. Discussions of the underlying mechanisms for the experimental phenomena are also provided based on kinematic analysis. The conclusions gained in this study can provide references for the optimization of machining parameters in 2D-UAG of hard and brittle materials.

## 1. Introduction

Silicon carbide (SiC) is extensively used in various industrial fields thanks to its outstanding performance of low density, high strength, and stable chemical properties [1]. However, due to its high hardness and low fracture toughness, the conventional grinding method generally faces challenges such as low efficiency, high cost, and poor machined surface quality [2,3]. Ultrasonic-assisted grinding (UAG) has been proven to be an effective machining method for hard and brittle materials with advantages of reducing grinding forces, improving surface quality, and inhibiting subsurface damages [4,5].

Recently, to meet the growing requirement for high-quality components made by difficult-to-machine material, the application of UAG in the field of material processing has attracted more attention, and numerous studies related to this hybrid processing method have been carried out. Dai et al. [6] conducted ultrasonic face grinding of SiC to investigate the effect of ultrasonic amplitude on the machined surface quality. The results showed that as the ultrasonic amplitude increased from 0 µm to 1 µm, the surface roughness firstly declined and then increased with a minimum value of 0.04 µm appearing at the amplitude of 0.216 µm. Yan et al. [7] conducted an axial ultrasonic-assisted grinding and scratching test of nano-ZrO_2_ ceramic to study the surface formation and damage mechanism during processing. The experimental results demonstrated that applying ultrasonic vibration can reduce the grinding force ratio and promote the propagation of lateral micro-cracks, thereby decreasing the subsurface damage depth. Zhao et al. [8] found that the subsurface crack form mainly contains arc-shaped cracks and bifurcated cracks in ultrasonic-assisted grinding of optical glass, and the ultrasonic amplitude has the greatest influence on the proportion of different cracks. Based on the above studies, to systematically reveal the influences of processing parameters on machined surface quality and provide an in-depth understanding of the surface generation mechanism in UAG of hard and brittle material, scholars in the field of material processing have established relevant theoretical models. Wu et al. [9] measured the protrusion high and vertex angle of abrasive grains on the grinding tool surface and further established a prediction model of surface roughness in the grinding of difficult-to-machine material, considering the material removal behaviors and machining parameters. This model was verified by grinding experiments of aluminum oxide ceramics and single crystal silicon with an average prediction error of 13%. For the face grinding of monocrystalline silicon, Li et al. [10] proposed a discrete numerical model to predict the surface roughness and provide a theoretical tool for the optimization of machining parameters. In the development of this model, the surface morphology of the grinding wheel was reconstructed, and the ductile-regime effect was considered through determining the grain–workpiece contact status. Based on the motion trajectory of abrasive grains and indentation fracture mechanics, Xiao et al. [11] described the material removal process during grinding and further proposed a prediction model of machined surface roughness with the consideration of all abrasive particles on the grinding wheel, which was verified by grinding experiments with an average error of 7.57%. Wen et al. [12] proposed a simplified model to describe rough surface profiles and investigated the influences of ultrasonic amplitude on the contact performance of machined surfaces in UAG. The results indicated that with increasing ultrasonic amplitude, the surface roughness of the machined surface first decreases and then increases, while the contact stiffness presents an opposite trend. The interference motion of abrasive grains contributes to a more concentrated surface height distribution, resulting in a better contact performance compared with that in conventional grinding. Despite the efforts made in the above studies, the contributions to the field of material processing are limited in quantifying the advantages of UAG in reducing grinding force and improving surface quality compared with conventional machining methods. The machining process was not classified into different stages according to the particle size of the grinding wheel, which is distinct from the industrial application. Therefore, the conclusions obtained in these studies have limited references for the manufacturing industry.

To further improve the machined surface quality of hard and brittle materials, scholars in this field adopted numerous optimization methods. On the surface of ordinary grinding wheels, the abrasive particles are randomly distributed with irregular protrusion heights, which is not conducive to improving the ground surface quality. Aiming at the above phenomenon, Ding et al. [13] proposed a brazed grinding wheel with uniform grain distribution and conducted UAG experiments of SiC using this grinding tool. The results demonstrated that under the same machining conditions, employing a brazed grinding wheel can effectively reduce the surface roughness compared with the electroplated one. Wen et al. [14] adopted surface-structured grinding wheels in conventional grinding of SiC and found that compared to a traditional grinding tool, a feather-like structured grinding wheel can reduce the normal and tangential forces by 56.3% and 47.7%, respectively. To improve the machining efficiency and machined surface quality of Reaction Bonded Silicon carbide (RB-SiC), Dong et al. [15] proposed a laser ablation-assisted ultrasonic grinding method, in which laser pre-ablation experiments were first conducted and the thermal damage layer was further removed by ultrasonic grinding. The results showed that this hybrid processing method can reduce the grinding force and surface roughness by 34% and 13.8% in comparison with conventional grinding. Qu and Pratap et al. [16,17] applied minimum quantity lubrication technology to the grinding of hard and brittle materials and found that this lubrication method can effectively reduce grinding force and surface roughness. For axial ultrasonic-assisted grinding, Wang et al. [18,19] proposed a matching model between ultrasonic vibration parameters and cutting parameters based on the kinematic characteristics, providing a theoretical tool to optimize the machining parameters. Depending on the experimental data of ultrasonic-assisted grinding of SiC, Lin et al. [20] established a one-dimensional fuzzy neural network surface roughness prediction model and further optimized the process parameters by employing a particle swarm algorithm, which provided a reference for actual production. However, in these studies, the influences of machining conditions on the surface quality and surface damage characteristics are not revealed in detail due to the lack of systematic machining experiments.

With the development of UAG, two-dimensional ultrasonic-assisted grinding (2D-UAG) has emerged and been applied in the machining of hard and brittle materials. By combining a rotary ultrasonic machining (RUM) device with a horizontally placed ultrasonic transducer, Wang et al. [21] proposed a 2D-UAG platform that can generate elliptical ultrasonic vibration. Theoretical analyses and 2D-UAG experiments of carbon fiber reinforced plastic (CFRP) were further conducted to reveal the effects of machining parameters on the grinding forces. Yan et al. [22] established a surface roughness prediction model for 2D-UAG of nano-ZrO2 ceramic, which was verified experimentally on a similar machining platform. This model provides a theoretical reference to evaluate the machined surface quality of hard and brittle materials. Ye et al. [23] conducted longitudinal torsional ultrasonic vibration grinding (LTUVG) of SiC and found that under the same machining conditions, LTUVG can reduce the surface roughness by 22.78% compared with conventional grinding. To avoid the severe surface damage in axial ultrasonic-assisted grinding of SiC, Cheng et al. [24] proposed a hybrid ultrasonic-assisted machining method involving axial vibration and elliptical vibration. The experimental results demonstrated that this novel machining method can effectively reduce the ground surface roughness by approximately 40.7% in comparison to traditional axial ultrasonic-assisted grinding. Through analysis of the literature, it is evident that the machining mechanism in 2D-UAG still has not been elaborately addressed, and the influences of two-dimensional ultrasonic vibrations on surface quality in different machining stages still remains unclear, resulting in a lack of theoretical basis for the optimization of machining parameters.

In this study, a 2D-UAG platform is established by involving the tangential vibration of workpiece and the axial vibration of grinding wheel, and the 2D-UAG grinding process are further divided into three stages according to the particle size of grinding wheel. To fill the above-mentioned gap, multi-step 2D-UAG experiments of SiC are conducted to systematically reveal the effects of machining parameters, including spindle speed, feed rate, grinding depth of cut, and ultrasonic amplitudes on the ground surface roughness and surface damage characteristics. Furthermore, discussions on the underlying mechanisms of the experimental results are also provided based on the previously proposed kinematic model. This paper is organized as follows: Section 2 describes experimental equipment and procedures, Section 3 presents the experimental results and corresponding discussions, and finally, conclusions are drawn in Section 4. The findings from this study offer practical references for the optimization of machining parameters in 2D-UAG of hard and brittle materials represented by SiC to improve machining quality and efficiency.

## 2. Experimental Procedures

### 2.1. Experimental Setup and Conditions

**Figure 1:**
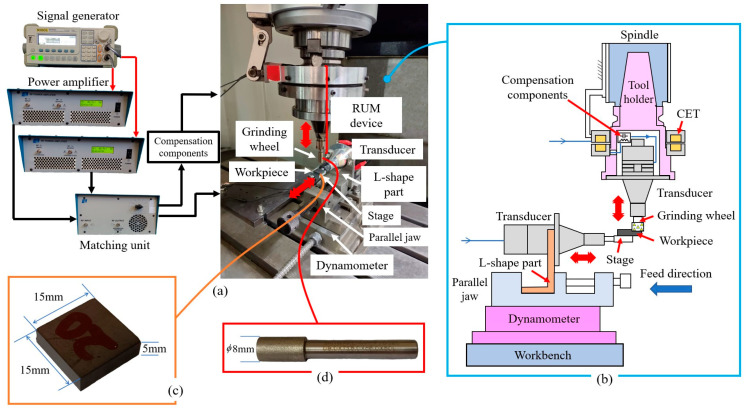
Schematic diagram of experimental apparatus: (**a**) detailed experimental setup; (**b**) 2D-UAG system; (**c**) workpiece; and (**d**) grinding wheel.

As illustrated in Figure 1, the 2D-UAG experiments are conducted on a G-VM5 CNC machine tool (Guangzhou Machine Tool Works, Guangzhou, China). The signal generator (G1022U, RIGOL, Suzhou, China), the power amplifier (1040 L, E&I, Anderson, SC, USA), and the matching unit (Lo-Hi-Z-8–500, E&I, USA) constitute an ultrasonic power supply to drive the machining device and realize the impedance matching between the power supply and the loads. The compensation components are employed to improve the transfer efficiency and power output capability of the contactless energy transfer (CET), which provides power for the revolving ultrasonic transducer. The RUM device can transmit axial ultrasonic vibration to the grinding wheel. With the assistance of an L-shaped part and a parallel jaw, the ultrasonic transducer can be placed horizontally, holding a stage at the output end. During machining, the workpiece is glued on the stage by epoxy resin and vibrates along the feed direction of the grinding wheel with this stage. Two channels of the signal generator are employed to produce sinusoidal signals with a frequency of 30 KHz to drive the RUM device and the ultrasonic transducer, respectively. The phase difference between the two channels is zero. Before experiments, a laser vibrometer (LV-S01-ST, Sunny Instruments, Singapore) is used to measure the vibration amplitudes of the grinding tool and workpiece, and the ultrasonic amplitudes can be manipulated by adjusting the driving voltage.

Electroplated diamond grinding wheels with a diameter of 8 mm are employed in 2D-UAG experiments, and the workpiece is pressureless silicon carbide with dimensions of 15 × 15 × 5 mm, as shown in Figure 1. The detailed material properties are provided in Table 1. According to the particle size of grinding wheel, the machining process is classified into three stages including rough grinding, semi-finishing grinding, and finishing grinding. To systematically reveal the influences of machining parameters on ground surface quality in each machining stage, the single-factor method is adopted to design experimental parameters, and detailed machining parameters are illustrated in Table 2.

### 2.2. Measurement and Evaluation

After experiments, the workpiece along with the stage are placed in an ultrasonic cleaning machine with acetone solution to dissolve the epoxy resin and remove chips. After that, the workpiece is cleaned again with purified water. A profilometer (IF G5, Alicona, Graz, Austria) is employed to obtain the surface roughness vertical to the feed direction. During evaluation, five regions on the ground surface are selected for measurement, and the average value of the five measurements is taken as the final result. Furthermore, a scanning electron microscope (JSM-IT300, JEOL, Akishima City, Tokyo, Japan) is used to observe the micro topographies of ground surfaces.

## 3. Results and Discussion

### 3.1. Effects of Spindle Speed on Surface Quality in Different Machining Stages

**Figure 2:**
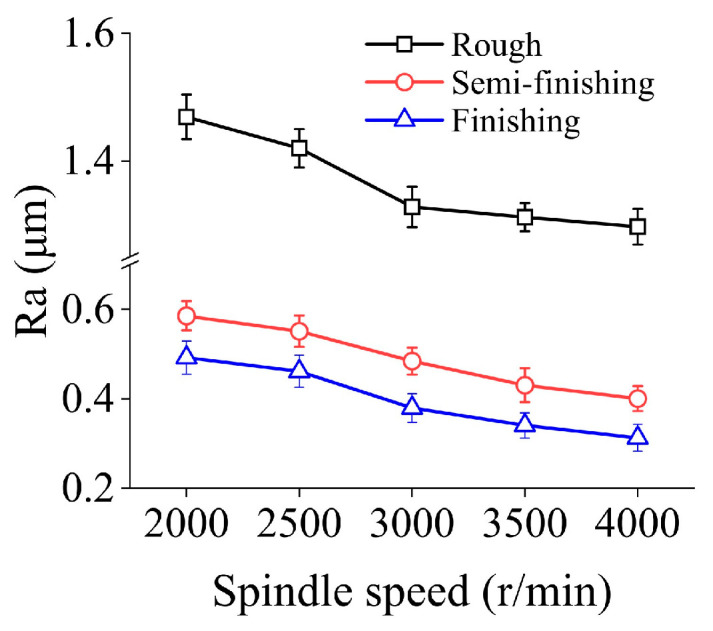
Surface roughness under different spindle speeds in three machining steps.

Figure 2 shows the influences of spindle speed on ground surface roughness in three machining stages. It can be found that as the spindle speed climbs from 2000 r/min to 4000 r/min, Ra presents a continuous decreasing trend under different particle sizes. In rough grinding, semi-finishing grinding, and finishing grinding, this indicator decreases by 11.71%, 31.57%, and 36.51%, respectively. In addition, when the spindle speed remains constant, the surface roughness decreases with the forward of the grinding stage, which indicates that the machining parameters selected in this study are reasonable.

**Figure 3:**
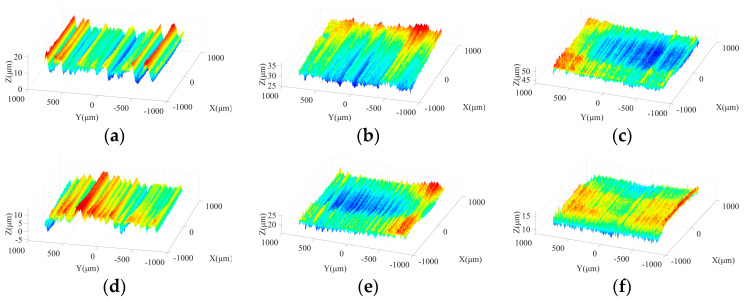
Machined surface morphologies under different spindle speeds in three grinding steps: (**a**) 240#, 2000 r/min; (**b**) 400#, 2000 r/min; (**c**) 600#, 2000 r/min; (**d**) 240#, 4000 r/min; (**e**) 400#, 4000 r/min; and (**f**) 600#, 4000 r/min.

Figure 3 shows the effects of spindle speed on ground surface morphology under different machining steps. As shown in Figure 3a,d, due to the large size and irregular protrusion height of abrasive grains in the rough grinding stage, the abrasive particles tend to leave grooves on the machined surface with considerable depths. With increasing spindle speed, the crest height and trough depth on the ground surface present a downward trend and grooves with a large depth are reduced, which is beneficial to improve the surface quality. In the semi-finishing grinding stage, resulting from the employment of the grinding wheel with a particle size of 400#, the average size and the difference among protrusion heights of abrasive grains are effectively reduced in comparison with those in the rough machining stage. Figure 3b,e indicate that a flatter ground surface with shallow grooves can be obtained in this step. As the spindle speed increases from 2000 r/min to 4000 r/min, the machined surface quality is further improved, attributed to the reduction of the maximum contour height and groove depth. Figure 3c,f show that the spindle speed affects the machined surface morphology under semi-finishing and finishing stages in a similar manner.

**Figure 4:**
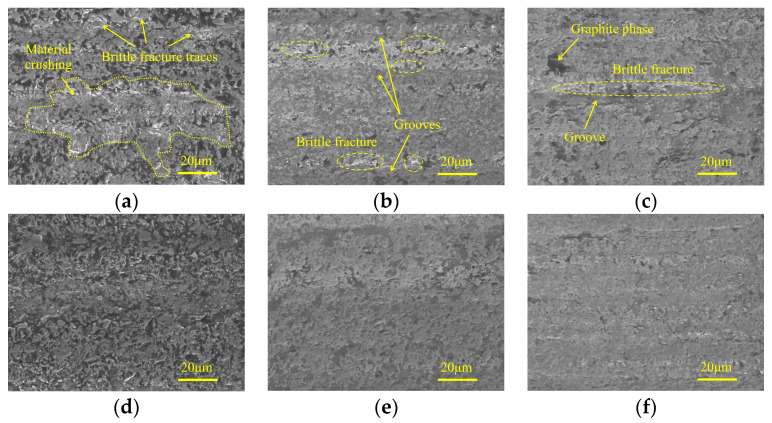
Micro-morphologies under different spindle speeds in three machining stages: (**a**) 240#, 2000 r/min; (**b**) 400#, 2000 r/min; (**c**) 600#, 2000 r/min; (**d**) 240#, 4000 r/min; (**e**) 400#, 4000 r/min; and (**f**) 600#, 4000 r/min.

Figure 4 shows the micro-morphologies of ground surfaces in three machining stages under different spindle speeds. Figure 4a,d indicate that in the rough grinding step, SiC is mainly removed by brittle fracture with obvious traces. When the spindle speed is 2000 r/min, material crushing with considerable area occurs due to the large, undeformed chip thickness of the individual active abrasive grain. With the increase of spindle speed, the material crushing phenomenon is effectively inhibited, and the machined surface is occupied by numerous irregular micro-pits, leading to an improved surface quality. In the semi-finishing machining stage, attributed to the increased particle size of the grinding wheel and reduced grinding depth of the cut and feed rate, the material ductile remove ratio is considerably increased, which is conducive to suppressing surface defects. When low spindle speed is adopted, grinding grooves with brittle fracture traces along the feed direction can be observed on the machined surface, forming damage regions with a strip distribution. An increase in spindle speed leads to a decrease of the above surface defects, resulting in a smooth and flat machined surface. In the finishing machining step, the influences of spindle speed on ground surface quality are similar to those in the semi-finishing step.

As other processing parameters remain constant, increasing the spindle speed effectively raises the cutting length of single abrasive grain, which further reduces the average cutting depth of individual particle at a constant total material removal rate. For hard and brittle materials represented by SiC, the above phenomenon is beneficial to suppress the adverse effects caused by inconsistent protrusion height of abrasive particles. On the other hand, increasing the spindle speed directly increases the cutting speed of active particles and elevates the strain rate in the material removal process, which can improve the dynamic fracture toughness of SiC and is conducive to inhibiting the generation and propagation of brittle cracks [25]. This factor is also one of the reasons for the above experimental phenomena.

### 3.2. Effects of Feed Rate on Surface Quality in Different Machining Stages

**Figure 5:**
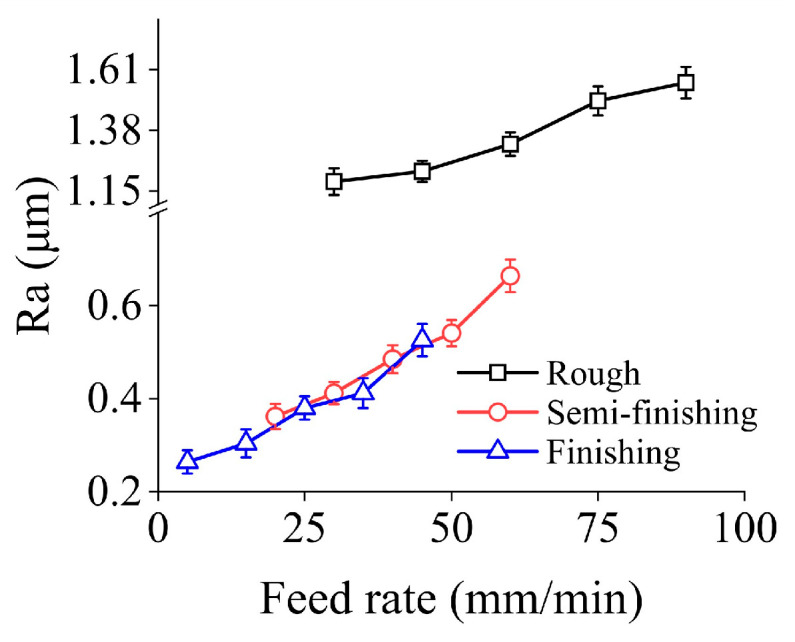
Surface roughness under different feed rates in three machining stages.

Figure 5 shows the effects of feed rate on the surface roughness in three machining stages. Due to the distinct process targets, the ranges of feed rate adopted under different grinding steps varies. It can be found that Ra presents an upward tendency with increasing feed rate in the parameter range discussed in this study. In the three machining steps, the increment of Ra is 31.62%, 83.43%, and 98.42%, respectively.

**Figure 6:**
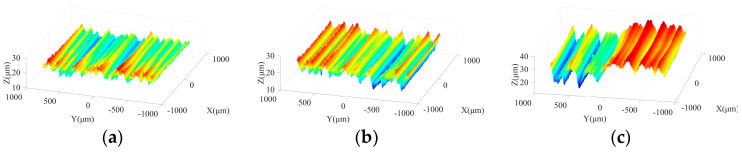
Surface morphologies under different feed rates in rough grinding step: (**a**) 240#, 30 mm/min; (**b**) 240#, 60 mm/min; and (**c**) 240#, 90 mm/min.

Figure 6 shows the ground surface morphologies under different feed rates in the rough grinding stage. It can be seen that when the feed rate is 30 mm/min, the grooves resulting from the interaction between abrasive particles and the workpiece are shallow, and the machined surface is relatively smooth and flat. With the increase of feed rate, the dimensions of grooves along with the maximum contour height grow, resulting in the deterioration of machined surface quality. The above phenomenon is consistent with the variation trend of surface roughness. Furthermore, in the semi-finishing and finishing stages, the effect of feed rate on surface quality is similar to that in the rough machining stage.

**Figure 7:**
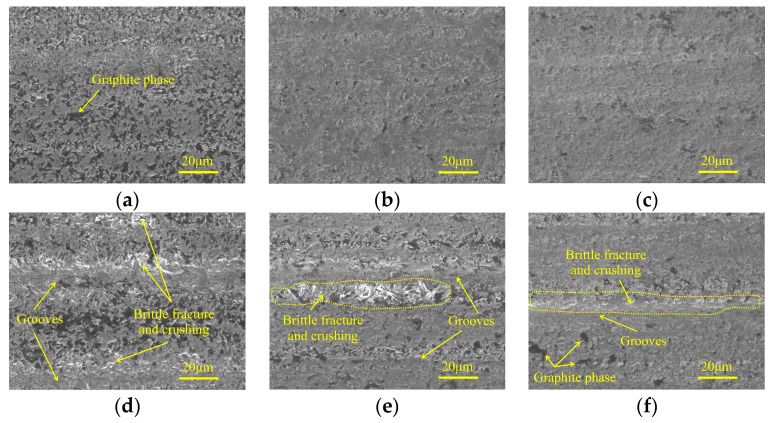
Micro-morphologies under different feed rates in three grinding stages: (**a**) 240#, 30 mm/min; (**b**) 400#, 20 mm/min; (**c**) 600#, 5 mm/min; (**d**) 240#, 90 mm/min; (**e**) 400#, 60 mm/min; and (**f**) 600#, 45 mm/min.

Figure 7 demonstrates the influences of feed rate on micro-morphologies of machined surfaces under different particle sizes. In the rough grinding step, as the feed rate climbs, the brittle fracture traces on ground surface become more obvious. When the feed rate is 90 mm/min, grooves with large depth are generated, and material fracture and crushing can be found at the edges of these trenches, leading to poor surface quality. In the semi-finishing and finishing grinding stages, the machined surface is flat and smooth under a low feed rate. With the growth of feed rate, shallow grooves and material fracture traces appear on the ground surface, forming damage regions with a stripe distribution along the feed direction and resulting in inferior processing quality.

In grinding process, when other processing parameters remain the same, an increase in feed rate directly enhances the material removal rate. On the other hand, keeping the grinding depth of the cut and spindle speed constant means that the contact length between the grinding wheel and workpiece and the number of effective abrasive grains passing through the machining area remain unchanged. Therefore, the average cutting length of individual abrasive particles is basically constant, while the maximum undeformed chip thickness increases with the growth of feed rate and material removal rate. In 2D-UAG of SiC, the above phenomenon generally causes more material to be removed by brittle fracture and increases the size of machining defects, leading to a deterioration of machined surface quality.

### 3.3. Effects of Grinding Depth of Cut on Surface Quality in Different Machining Stages

**Figure 8:**
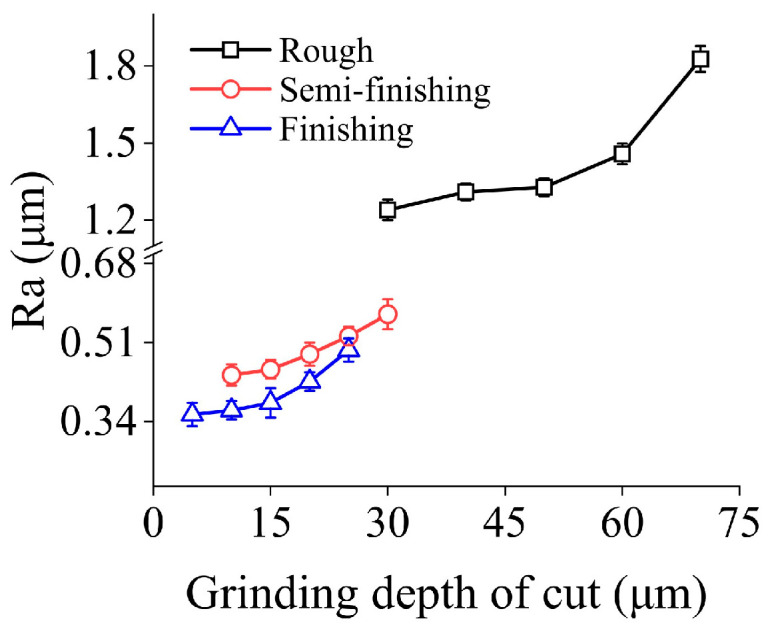
Surface roughness under different grinding depth of cut in three machining steps.

Figure 8 demonstrates the effects of grinding depth of cut on surface roughness under different machining steps. It can be found that within the range of machining parameters discussed, Ra obtained in three machining stages under conservative grinding depth of cut is 1.24 μm, 0.44 μm, and 0.35 μm, respectively. With increasing grinding depth of cut, Ra shows a monotonically growth tendency with increments of 47.34%, 29.77%, and 41.14% in different grinding stages.

**Figure 9:**
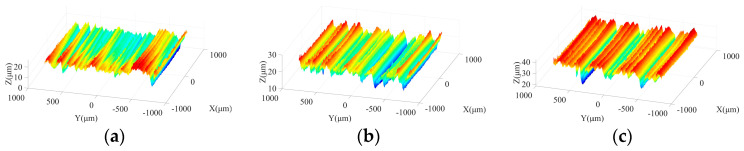
Surface morphologies under different grinding depth of cut in rough machining step: (**a**) 240#, 30 μm; (**b**) 240#, 50 μm; and (**c**) 240#, 70 μm.

Figure 9 shows the ground surface morphologies under different grinding depth of cut in the rough grinding step. It demonstrates that when the grinding depth of cut is 30 μm, the machined surface is flat, and shallow grinding grooves parallel to the feed direction can be observed. The elevation in grinding depth of cut leads to the growth of the maximum contours height and the width of grinding trenches, resulting in a poor surface quality. In the semi-finishing and finishing grinding steps, the influences of grinding depth of cut on the machined surface morphology are similar to those in rough grinding.

**Figure 10:**
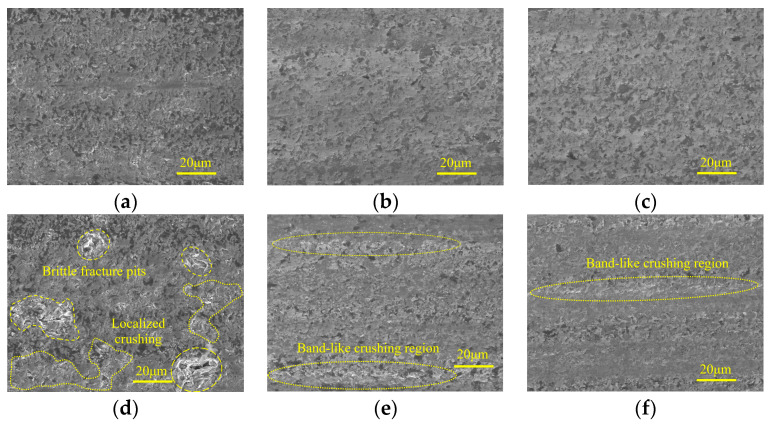
Micro-morphologies under different grinding depth of cut in three machining steps: (**a**) 240#, 30 μm; (**b**) 400#, 10 μm; (**c**) 600#, 5 μm; (**d**) 240#, 70 μm; (**e**) 400#, 30 μm; and (**f**) 600#, 25 μm.

Figure 10 presents the micro-morphologies of machined surfaces under different grinding depth of cut in three grinding steps. It can be found that, regardless of the particle size, the machined surface quality degrades with increasing grinding depth of cut. In the rough grinding step, when the grinding depth of cut is 70 μm, the surface defects mainly contain localized material crushing and pits caused by brittle fracture. In the semi-finishing and finishing machining stages, the degree of surface quality deterioration is not significant. In the former step, adopting greater grinding depth of cut generally results in the appearance of material crushing regions with a band-like distribution, which consisted of small-sized brittle fracture traces. In the latter machining stage, a similar phenomenon can be observed, and the deterioration in surface quality is further suppressed.

In the grinding process, increasing the grinding depth of cut is beneficial to improve the material removal rate and increase the contact area between the grinding wheel and the workpiece, which further enhances the contact length and extends the effective cutting time of individual active grain. A previous study [26] showed that in 2D-UAG, the instantaneous cutting depth of active abrasive grits with a large protrusion height fluctuates periodically when passing through the machining area, and the average cutting depth within a single fluctuation cycle gradually increases with grinding time. Therefore, with the growth of grinding depth of cut, the increase in cutting time raises the maximum cutting depth of active grits in a single spindle rotation cycle, which will further result in poor machining quality. On the other hand, when large particle size and grinding depth of cut are used, the chips generated during material removal are difficult to discharge from the grinding area. This phenomenon generally results in an accumulation of chips on the grinding wheel surface and the ground surface, which is also not conducive to improving the machining quality.

### 3.4. Effects of Ultrasonic Vibrations on Surface Quality in Different Machining Stages

**Figure 11:**
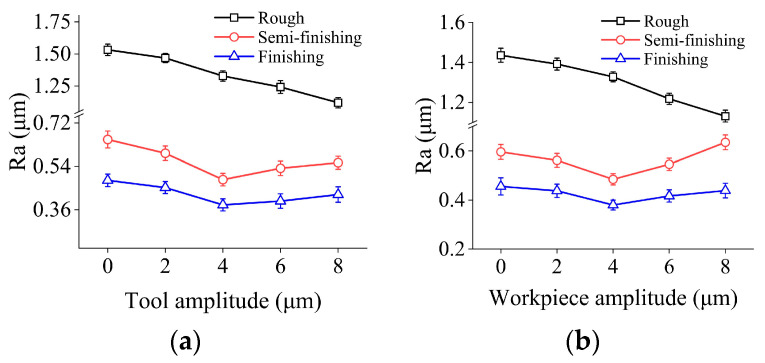
Surface roughness under different ultrasonic amplitudes in three machining steps: (**a**) effects of tool amplitude; and (**b**) effects of workpiece amplitude.

Figure 11 presents the influences of tool vibration and workpiece vibration on surface roughness in three grinding stages. It can be found that in the rough machining stage, Ra shows a monotonically decreasing trend with a maximum reduction of 26.9% when the tool amplitude increases from 0 μm to 8 μm. The above data suggest that increasing the tool amplitude is beneficial to reduce the number of crests and troughs on the machined surface and flattens the height distribution, which consequently improves the ground surface quality. Furthermore, Ra also decreases monotonically as the workpiece amplitude increases. Specifically, Ra reduces by 21.24% when the workpiece amplitude grows from 0 μm to 8 μm.

In the semi-finishing machining step, the grinding wheel with a particle size of 400# is employed. As the tool amplitude increases from 0 μm to 4 μm, Ra presents a continuous downward trend with a maximum drop of 25.5%. However, when the tool amplitude is further increased from 4 μm to 8 μm, this indicator increases by 14.43%. On the other hand, as the workpiece amplitude grows from 0 μm to 8 μm, a similar phenomenon can be observed. It is worth noting that when the workpiece amplitude increases from 4 μm to 8 μm, Ra has a greater increment of 30.93% in comparison with that of the tool amplitude. In the finishing machining stage, the grinding wheel with a particle size of 600# is used and the ultrasonic amplitudes affect the machined surface roughness in a similar manner with that in semi-finishing machining stage. Furthermore, when the tool amplitude and workpiece amplitude remain the same, the Ra obtained in the roughing, semi-finishing, and finishing stages decreases sequentially.

**Figure 12:**
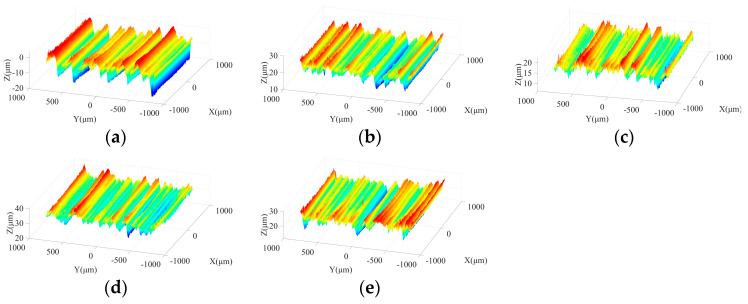
Surface morphologies under different ultrasonic amplitudes in rough grinding stage: (**a**) $T_{A}$: 0 μm $W_{A}$: 4 μm; (**b**) $T_{A}$: 4 μm $W_{A}$: 4 μm; (**c**) $T_{A}$: 8 μm $W_{A}$: 4 μm; (**d**) $W_{A}$: 0 μm $T_{A}$: 4 μm; and (**e**) $W_{A}$: 8 μm $T_{A}$:4 μm.

Figure 12 shows the ground surface morphologies under different ultrasonic amplitudes in the rough machining stage. As illustrated in Figure 12a–c, when the tool amplitude is 0 μm, obvious grinding traces are distributed on the machined surface. With the growth of the tool amplitude, the maximum contour height of the ground surface is effectively reduced and the spacing of wave crests decreases, leading to an improved surface quality. Figure 12b,d,e demonstrates that with the increase of workpiece amplitude, a similar phenomenon can be observed. Meanwhile, the comparison between Figure 12a,d shows that when the tool amplitude or the workpiece amplitude is 0 μm, superior surface quality with lower crest heights and trough depths can be obtained in the latter machining conditions. A possible reason is that under the latter working condition, the vibration of the grinding wheel in the axial direction during processing effectively prolongs the motion trajectory of active abrasive grains and enhances the overlapping phenomena of these trajectories, which strengthens the interaction among active abrasive particles and further contributes to the improvement of surface quality. As shown in Figure 12c,e, when the workpiece amplitude is increased to 8 μm, troughs with large depth can be observed on the localized machined surface, which may be caused by the impact behaviors of active abrasive grits on the machined area. Meanwhile, the above phenomenon is alleviated when the tool amplitude is increased to the same level.

**Figure 13:**
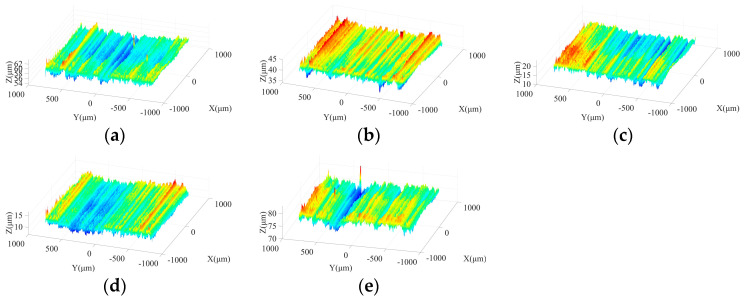
Surface morphologies under different ultrasonic amplitudes in semi-finishing grinding stage: (**a**) $T_{A}$: 0 μm $W_{A}$: 4 μm; (**b**) $T_{A}$: 4 μm $W_{A}$: 4 μm; (**c**) $T_{A}$: 8 μm $W_{A}$: 4 μm; (**d**) $W_{A}$: 0 μm $T_{A}$: 4 μm; and (**e**) $W_{A}$: 8 μm $T_{A}$: 4 μm.

**Figure 14:**
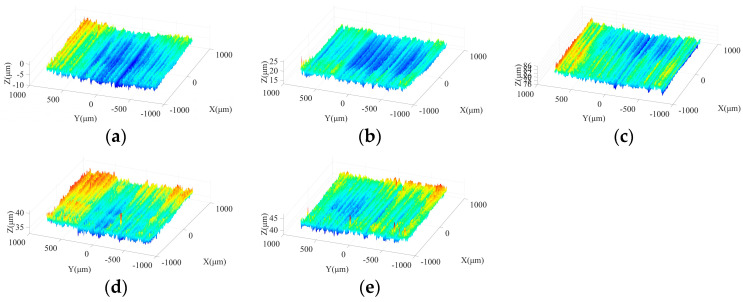
Surface morphologies under different ultrasonic amplitudes in finishing grinding stage: (**a**) $T_{A}$: 0 μm $W_{A}$: 4 μm; (**b**) $T_{A}$: 4 μm $W_{A}$: 4 μm; (**c**) $T_{A}$: 8 μm $W_{A}$: 4 μm; (**d**) $W_{A}$: 0 μm $T_{A}$: 4 μm; and (**e**) $W_{A}$: 8 μm $T_{A}$: 4 μm.

Figure 13 shows the morphologies of machined surfaces under different ultrasonic amplitudes in semi-finishing stage. It indicates that with the increase in particle size, the ground surface quality is effectively improved compared to that obtained in the roughing stage. When the tool amplitude or workpiece amplitude increases from 0 μm to 4 μm, the maximum contour height of ground surfaces decreases, and flatter surface topography can be obtained. However, as the ultrasonic amplitude is further increased from 4 μm to 8 μm, the crest height and trough depth on the machined surface increases again, resulting in a deterioration of surface quality. Meanwhile, as presented in Figure 13a,d, when only one-dimensional ultrasonic vibration is applied, axial tool vibration is beneficial to avoid the appearance of grinding grooves with large depth on the machined surface compared to tangential workpiece vibration, contributing to the improvement of surface quality. Similar to the roughing stage, the comparison between Figure 13c,e shows that when a large workpiece amplitude is adopted, the depth of partial troughs on the machined surface increases due to the impact behaviors of active grains, which further leads to the surface quality degradation. Therefore, the selection of excessive workpiece amplitude should be avoided in the machining process. In addition, Figure 14 demonstrates that the influences of ultrasonic amplitudes on the machined surface morphologies in the finishing grinding stage are similar with those in the semi-finishing step.

**Figure 15:**
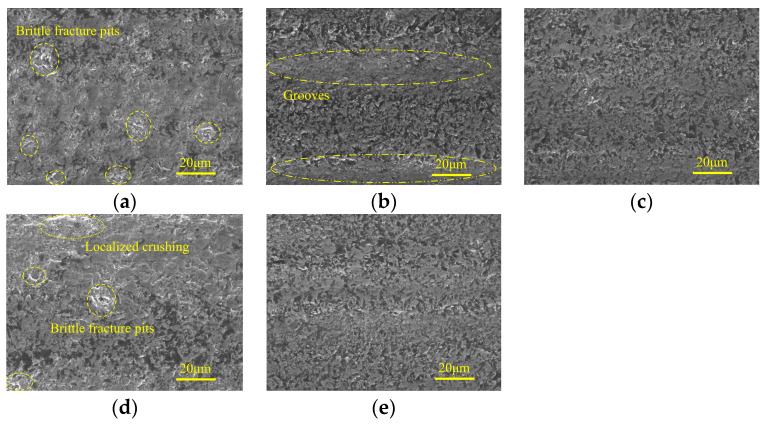
Micro-morphologies under different ultrasonic amplitudes in rough grinding stage: (**a**) $T_{A}$: 0 μm $W_{A}$: 4 μm; (**b**) $T_{A}$: 4 μm $W_{A}$: 4 μm; (**c**) $T_{A}$: 8 μm $W_{A}$: 4 μm; (**d**) $W_{A}$: 0 μm $T_{A}$: 4 μm; and (**e**) $W_{A}$: 8 μm $T_{A}$: 4 μm.

The micro-morphologies of machined surfaces obtained in the rough grinding stage under different ultrasonic amplitudes are shown in Figure 15. It indicates that when minor tool amplitude or workpiece amplitude is used, the brittle fracture traces along with large area material crushing are obvious on machined surfaces. Meanwhile, irregular pits with large depth are distributed on localized ground surfaces, which may be resulted from the propagation of brittle cracks to internal defects in the workpiece. As the tool amplitude or workpiece amplitude increases, the surface quality is improved, and the ground surface is occupied by discrete micro-pits with shallow depth. Simultaneously, grooves generated by abrasive grains with high protrusion heights can be observed on the ground surface, and the bottom of these grooves is relatively flat and smooth, while localized material crushing can be observed at the edges.

**Figure 16:**
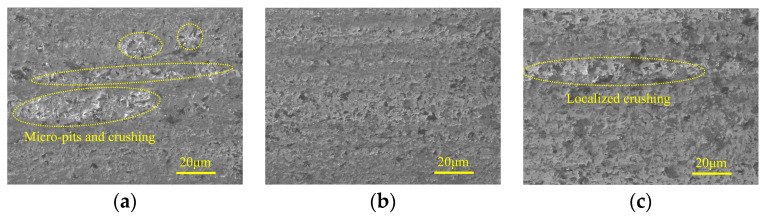

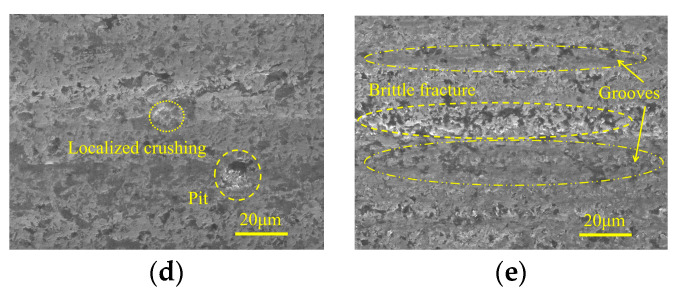
Micro-morphologies under different ultrasonic amplitudes in semi-finishing grinding: (**a**) $T_{A}$: 0 μm $W_{A}$: 4 μm; (**b**) $T_{A}$: 4 μm $W_{A}$: 4 μm; (**c**) $T_{A}$: 8 μm $W_{A}$: 4 μm; (**d**) $W_{A}$: 0 μm $T_{A}$: 4 μm; and (**e**) $W_{A}$: 8 μm $T_{A}$: 4 μm.

**Figure 17:**
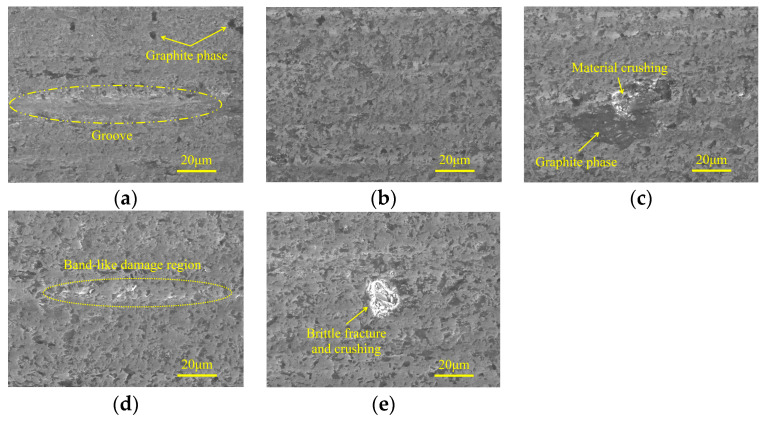
Micro-morphologies under different ultrasonic amplitudes in finishing grinding stage: (**a**) $T_{A}$: 0 μm $W_{A}$: 4 μm; (**b**) $T_{A}$: 4 μm $W_{A}$: 4 μm; (**c**) $T_{A}$: 8 μm $W_{A}$: 4 μm; (**d**) $W_{A}$: 0 μm $T_{A}$: 4 μm; and (**e**) $W_{A}$: 8 μm $T_{A}$: 4 μm.

Figure 16 shows the micro-morphologies of machined surfaces under different ultrasonic amplitudes in the semi-finishing machining stage. It demonstrates that when the tool amplitude is 0 μm, despite the fact that the majority of the machined surface is relatively flat and smooth, micro-pits and material crushing still remain in localized areas, forming damage regions with a band-like distribution. As the tool amplitude increases from 0 μm to 8 μm, the surface quality is first improved and then deteriorates with an inflection point at 4 μm. As shown in Figure 16b,d,e, the effects of tool amplitude and workpiece amplitude on the micro-morphologies of machined surfaces are similar. When the workpiece amplitude is increased to 8 μm, the surface defect mainly contains grinding grooves, and the edges of these grooves are accompanied by brittle fracture traces. Figure 17 shows the effects of ultrasonic amplitudes on micro-morphologies of machined surfaces under finishing machining stage and indicates that in the machining parameter ranges discussed in this study, moderate ultrasonic amplitudes are beneficial to improve ground surface quality. When minor ultrasonic amplitudes are employed, the surface defects are mainly composed of localized grooves and band-like damage regions. Meanwhile, when the tool amplitude or workpiece amplitude is large, brittle fracture and material crushing with considerable area are prone to occur around the graphite phase.

**Figure 18:**
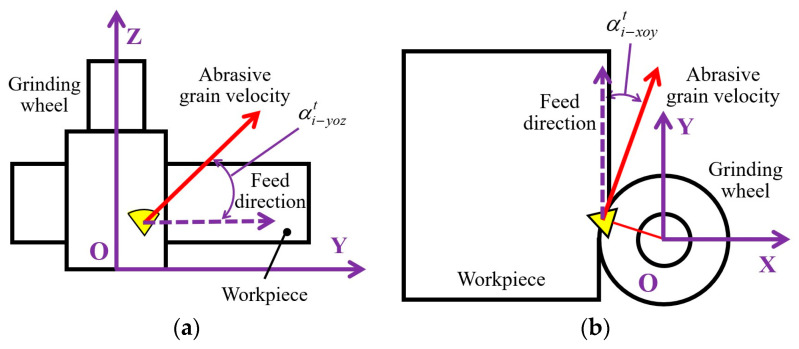
Definitions of cutting angles [26]: (**a**) in XOY plane; and (**b**) in YOZ plane.

**Figure 19:**
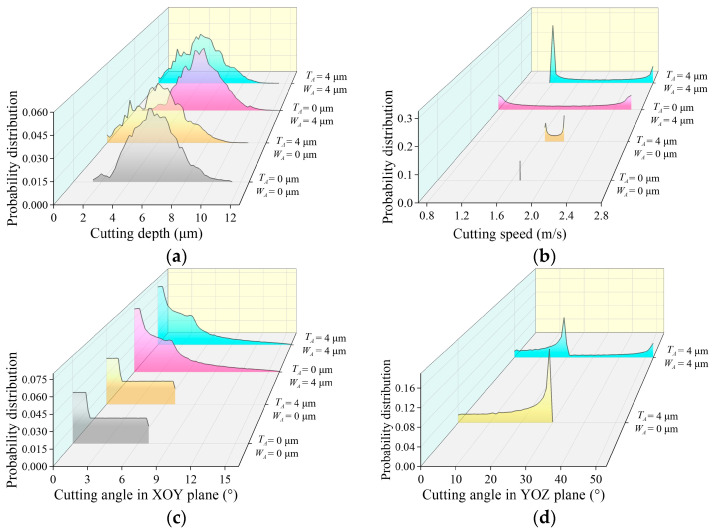
Influences of ultrasonic amplitudes on kinematic features of active abrasive grains [26]: (**a**) the distribution of cutting depth; (**b**) the distribution of cutting speed; (**c**) the distribution of the cutting angle in XOY plane; (**d**) the distribution of the cutting angle in YOZ plane.

In previous research, a discrete numerical model was proposed for 2D-UAG of SiC to describe the dynamic cutting behaviors of active abrasive grains [26,27]. To characterize the cutting direction of abrasive particles with respect to the workpiece, the XOY plane cutting angle $\alpha_{i-xoy}^{t}$ and YOZ plane cutting angle $\alpha_{i-yoz}^{t}$ are defined, as shown in Figure 18. By discretizing the grinding wheel and workpiece into a series of feature points, this model is capable of obtaining detailed kinematic information of active grits in terms of cutting depth, cutting speed, and the above-mentioned cutting angles based on grinding kinematics. In the rough machining stage, resulting from the particle size of the grinding wheel and the machining parameters adopted, the workpiece material is mainly removed by brittle fracture, leading to a poor surface quality. As shown in Figure 19, the above theoretical model indicates that the application of tool vibration in 2D-UAG is beneficial to reduce the average cutting depth of active grains, which further increases the ductile removal rate of workpiece material. On the other hand, the superposition of tool vibration and workpiece vibration effectively expands the distribution ranges of cutting angles in the XOY plane and YOZ plane of active grains, which is conducive to promoting the propagation of brittle cracks along multiple directions and further strengthening the interference among cracks. The above phenomena are beneficial to reduce the size of brittle fracture and inhibit the adverse effects of inconsistent protrusion height of abrasive grains. Meanwhile, the elevation of ultrasonic amplitudes increases the maximum cutting speed of abrasive particles, which can improve the dynamic fracture toughness of SiC and suppress the emergence and expansion of brittle cracks. Therefore, the machined surface quality in the rough grinding stage gradually improves with the increase of tool amplitude or workpiece amplitude. Furthermore, in this machining step, the material is removed mainly by brittle fracture, leading to the impact behaviors of abrasive particles under considerable workpiece amplitude on the workpiece having a limited influences on ground surface quality.

In semi-finishing and finishing grinding steps, resulting from the adjustment of the wheel grain size, feed rate, and grinding depth of cut, the proportion of material ductile removal increases significantly, leading to an improved machined surface quality. Theoretical analysis indicates that in 2D-UAG, when the tool amplitude remains constant, the distribution ranges of cutting angles in XOY and YOZ planes are enlarged with the increase of workpiece amplitude, which is beneficial to improve the machined surface quality. Therefore, as the workpiece amplitude grows from 0 μm to 4 μm, the ground surface quality gradually advances. However, due to the presence of workpiece vibration, the active abrasive grits with lower protrusion heights enter an intermittent cutting state, meaning that these abrasive particles are only partially involved in material removal during a single vibration cycle. With the further increase of workpiece amplitude, more abrasive grains enter the intermittent cutting state, which leads to a variation in the number of active grits and increases the maximum distribution limit of cutting depth. Meanwhile, the impact behavior of abrasive grains on the workpiece continues to enhance with the increase in workpiece amplitude. As a result, a deterioration of the machined surface quality occurs at semi-finishing and finishing grinding stages when the workpiece amplitude is further increased from 4 μm to 8 μm.

Under the working conditions discussed in this study, when the workpiece amplitude remains constant, the distribution range of the cutting angle in the YOZ plane gradually expands and the overlapping phenomenon among trajectories of active grits is enhanced as the tool amplitude increases, leading to a decrease in the average cutting depth of individual grain. Simultaneously, the interference behaviors among brittle cracks are strengthened. Therefore, the increase of tool amplitude from 0 μm to 4 μm contributes to the improvement of machined surface quality. However, for the abrasive grains in intermittent cutting state, the increase of tool amplitude will enhance the cutting length or cutting width in a single vibration cycle. Furthermore, with the increase of ultrasonic amplitude, the impact of these active grains on the workpiece is also more significant. The above phenomena are the possible reasons for the deterioration of machined surface quality when the tool amplitude is further increased to 8 μm.

## 4. Conclusions

In this paper, multi-step 2D-UAG experiments of SiC are conducted to reveal the influences of cutting parameters, tool amplitude, workpiece amplitude, and wheel grain size on the ground surface quality. Discussions of the underlying mechanisms for the above experimental phenomena are also provided combined with the kinematic analysis to lay a foundation for the selection and optimization of machining parameters. Key findings include:

(1) In 2D-UAG of SiC, when other machining parameters remain constant, the Ra value decreases with the growth of spindle speed and increases with increasing feed rate or grinding depth of cut. In different machining stages, the tool amplitude and workpiece amplitude affect the surface roughness in a similar manner. Under the rough grinding step, Ra presents a continuous downward tendency with the increase of ultrasonic amplitudes. Meanwhile, in the semi-finishing or finishing grinding step, Ra first decreases and then increases with the growth of ultrasonic amplitudes. Within the range of processing parameters discussed in this study, the inflection point appears around 4 μm.

(2) In the rough grinding stage, when the cutting parameters are identical, the surface roughness obtained by 2D-UAG reduces 7.52% and 13.32% in comparison with that gained in axial ultrasonic-assisted grinding and tangential ultrasonic-assisted grinding under an ultrasonic amplitude of 4 μm. Similar phenomena can be observed in semi-finishing and finishing grinding steps.

(3) The three-dimensional morphologies of ground surfaces show that due to the inconsistent protrusion height of abrasive grains, grooves with various depth parallel to the feed direction are generated on machined surfaces. In three machining stages, the reasonable adjustment of cutting parameters and ultrasonic parameters can effectively reduce the depth and width of the grooves, further improving the machined surface quality.

(4) The micro-morphologies of machined surfaces demonstrate that in the rough grinding stage, SiC is mainly removed by brittle fracture and the surface defects contain large-size brittle fracture pits, material crushing, and localized grooves, which constitute damage regions. In the semi-finishing and finishing grinding steps, the ductile material removal ratio increases, leading to an improved surface quality. The surface defect is mainly composed of small-size pits and localized grooves, forming band-like damage areas. Furthermore, localized material crushing is prone to occur around the graphite phase when large ultrasonic amplitudes are adopted.

## Figures and Tables

**Table 1:** Material properties.

Hardness (GPa)	Density (g/cm^3^)	Elastic Modulus (GPa)	Average Particle Size (μm)	Fracture Toughness (MPa·m^1/2^)	Bending Strength (GPa)
27.7	3.15	410	4–10	4.7	0.38

**Table 2:** Machining parameters.

No.	Spindle Speed (r/min)	Feed Rate (mm/min)	Grinding Depth of Cut (μm)	Workpiece Amplitude $W_{A}$ (μm)	Tool Amplitude $T_{A}$ (μm)	Grit Size (#)
1	2000, 2500, 3000, 3500, 4000	30, 45, 60, 75, 90	30, 40, 50, 60, 70	0, 2, 4, 6, 8	0, 2, 4, 6, 8	240
2	2000, 2500, 3000, 3500, 4000	20, 30, 40, 50, 60	10, 15, 20, 25, 30	0, 2, 4, 6, 8	0, 2, 4, 6, 8	400
3	2000, 2500, 3000, 3500, 4000	5, 15, 25, 35, 45	5, 10, 15, 20, 25	0, 2, 4, 6, 8	0, 2, 4, 6, 8	600

## Data Availability

Data will be made available on request.

## References

[1] Khodaei M., Yaghobizadeh O., Alhosseini S.H.N., Esmaeeli S., Mousavi S.R. (2019). The effect of oxide, carbide, nitride and boride additives on properties of pressureless sintered SiC: A review. J. Eur. Ceram. Soc..

[2] Huang C., Zhou M., Zhang H. (2022). Investigations on the micro-interactions of grit-workpiece and forces prediction in ultrasonic vibration side grinding of optical glass. J. Mater. Process. Technol..

[3] Huang C., Zhou M., Zhang H. (2021). A cutting force prediction model in axial ultrasonic vibration end grinding for BK7 optical glass considering protrusion height of abrasive grits. Measurement.

[4] Yang Z., Zhu L., Zhang G., Ni C., Lin B. (2020). Review of ultrasonic vibration-assisted machining in advanced materials. Int. J. Mach. Tools Manuf..

[5] Ning F., Cong W. (2020). Ultrasonic vibration-assisted (UV-A) manufacturing processes: State of the art and future perspectives. J. Manuf. Process..

[6] Dai C., Yin Z., Wang P., Miao Q., Chen J. (2021). Analysis on ground surface in ultrasonic face grinding of silicon carbide (SiC) ceramic with minor vibration amplitude. Ceram. Int..

[7] Yan Y., Zhang Y., Zhao B., Liu J. (2021). Surface formation and damage mechanisms of nano-ZrO2 ceramics under axial ultrasonic-assisted grinding. J. Mech. Sci. Technol..

[8] Zhao P., Zhang L., Liu X. (2021). Crack Forms Sensitivity-Based Prediction on Subsurface Cracks Depth in Ultrasonic-Vibration-Assisted Grinding of Optical Glasses. Appl. Sci..

[9] Wu J., Cheng J., Gao C., Yu T., Guo Z. (2020). Research on predicting model of surface roughness in small-scale grinding of brittle materials considering grinding tool topography. Int. J. Mech. Sci..

[10] Li H.N., Yu T.B., Zhu L.D., Wang W.S. (2017). Analytical modeling of ground surface topography in monocrystalline silicon grinding considering the ductile-regime effect. Arch. Civ. Mech. Eng..

[11] Xiao X., Li G., Li Z. (2021). Prediction of the Surface Roughness in Ultrasonic Vibration-Assisted Grinding of Dental Zirconia Ceramics Based on a Single-Diamond Grit Model. Micromachines.

[12] Wen Y., Tang J., Zhou W., Zhu C. (2019). Study on contact performance of ultrasonic-assisted grinding surface. Ultrasonics.

[13] Ding K., Li Q., Lei W., Zhang C., Xu M., Wang X. (2022). Design of a defined grain distribution brazed diamond grinding wheel for ultrasonic assisted grinding and experimental verification. Ultrasonics.

[14] Wen D., Wan L., Zhang X., Li C., Ran X., Chen Z. (2023). Grinding performance evaluation of SiC ceramic by bird feather-like structure diamond grinding wheel. J. Manuf. Process..

[15] Dong Z., Sun W., Cai X., Ding K., Bao Y., Ma G., Wu D., Kang R., Niu F. (2023). A new method for machining RB-SiC with high efficiency and quality: Laser assisted ultrasonic grinding. J. Manuf. Process..

[16] Choudhary A., Paul S. (2020). The wear mechanisms of diamond grits in grinding of alumina and yttria-stabilized zirconia under different cooling-lubrication schemes. Wear.

[17] Pratap A., Patra K. (2020). Combined effects of tool surface texturing, cutting parameters and minimum quantity lubrication (MQL) pressure on micro-grinding of BK7 glass. J. Manuf. Process..

[18] Wang Y., Lin B., Cao X., Wang S. (2014). An experimental investigation of system matching in ultrasonic vibration assisted grinding for titanium. J. Mater. Process. Technol..

[19] Wang Y., Lin B., Wang S., Cao X. (2014). Study on the system matching of ultrasonic vibration assisted grinding for hard and brittle materials processing. Int. J. Mach. Tools Manuf..

[20] Lin C., Jhang J., Young K. (2020). Parameter Selection and Optimization of an Intelligent Ultrasonic-Assisted Grinding System for SiC Ceramics. IEEE Access.

[21] Wang H., Pei Z.J., Cong W. (2020). A feeding-directional cutting force model for end surface grinding of CFRP composites using rotary ultrasonic machining with elliptical ultrasonic vibration. Int. J. Mach. Tools Manuf..

[22] Yan Y., Zhang Z., Zhao B., Liu J. (2021). Study on prediction of three-dimensional surface roughness of nano-ZrO_2_ ceramics under two-dimensional ultrasonic-assisted grinding. Int. J. Adv. Manuf. Technol..

[23] Ye Z., Wen X., Wan W., Liu F., Bai W., Xu C., Chen H., Gong P., Han G. (2023). Precision Grinding Technology of Silicon Carbide (SiC) Ceramics by Longitudinal Torsional Ultrasonic Vibrations. Materials.

[24] Cheng Q., Dai C., Miao Q., Yin Z., Chen J., Yang S. (2023). Axial and composite ultrasonic vibration-assisted face grinding of silicon carbide ceramics: Grinding force and surface quality. Int. J. Adv. Manuf. Technol..

[25] Wu C., Li B., Liu Y., Liang S.Y. (2017). Surface roughness modeling for grinding of Silicon Carbide ceramics considering co-existence of brittleness and ductility. Int. J. Mech. Sci..

[26] Li H., Chen T., Duan Z., Zhang Y., Li H. (2023). Analytical and experimental study on the surface generation mechanism in two-dimensional ultrasonic-assisted grinding of silicon carbide. Int. J. Adv. Manuf. Technol..

[27] Li H., Chen T., Duan Z., Zhang Y., Li H. (2022). A grinding force model in two-dimensional ultrasonic-assisted grinding of silicon carbide. J. Mater. Process. Technol..

